# Emerging role of ferroptosis-related circular RNA in tumor metastasis

**DOI:** 10.3389/fphar.2023.1168458

**Published:** 2023-04-24

**Authors:** Yifei Meng, Jingdong Cao, Yidan Li, Saili Duan, Zongjiang Zhou, Jinghe Li, Diabate Ousmane, Chunlin Ou, Junpu Wang

**Affiliations:** ^1^ Department of Pathology, Xiangya Hospital, Central South University, Changsha, China; ^2^ Department of Pathology, School of Basic Medicine, Central South University, Changsha, China; ^3^ Department of Pathology, Ultrapathology (Biomedical Electron Microscopy) Center, Xiangya Hospital, Central South University, Changsha City, China; ^4^ Key Laboratory of Hunan Province in Neurodegenerative Disorders, Xiangya Hospital, Central South University, Changsha, China; ^5^ National Clinical Research Center for Geriatric Disorders, Xiangya Hospital, Central South University, Changsha, China

**Keywords:** tumor metastasis, CircRNAs, ferroptosis, biomarkers, therapeutic target

## Abstract

Tumor metastasis is an important factor that contributes to the poor prognosis of patients with tumors. Therefore, to solve this problem, research on the mechanism of metastasis is essential. Ferroptosis, a new mode of cell death, is characterized by membrane damage due to lipid peroxidation caused by iron overload. Many studies have shown that excessive ferroptosis can affect tumor metastasis and thus inhibit tumor progression. Recently, circular RNA (circRNA), a type of non-coding RNA, has been shown to be associated with the progression of ferroptosis, thus influencing tumor development. However, the specific mechanisms by which circRNAs affect the progression of ferroptosis and their roles in tumor metastasis are not known. In this review, we systematically discuss the role of circRNAs in regulating tumor ferroptosis and their mechanism of action through sponging miRNAS in various tumors, thereby impacting metastasis. This review helps elucidate the relationship and role of ferroptosis-related circRNAs in tumor metastasis and may provide future researchers with new ideas and directions for targeted therapies.

## 1 Introduction

Tumor metastasis is strongly associated with patient prognosis, contributing to more than 90% of cancer-related deaths (Dykes et al., 2019; Jakoš, 2019). Compared to limited primary tumors with good prognoses, metastases can cause complex systemic diseases due to the systemic spread of disseminated and circulating tumor cells, making existing treatment measures, such as surgical resection, less effective (Fu, 2020). Therefore, studying the mechanisms of metastasis and related molecules is beneficial for identifying detection indicators and therapeutic targets. It helps to develop targeted preventive and therapeutic measures to improve the prognosis of patients with cancer (Psaila and Lyden, 2009; Peinado et al., 2017; Coban et al., 2021; Novikov et al., 2021). Many studies have focused on the role of circular RNAs (circRNAs) and ferroptosis in tumor progression; however, the relationship between the two is not well understood. We briefly introduce both concepts and their links to tumor progression.

**FIGURE 1:**
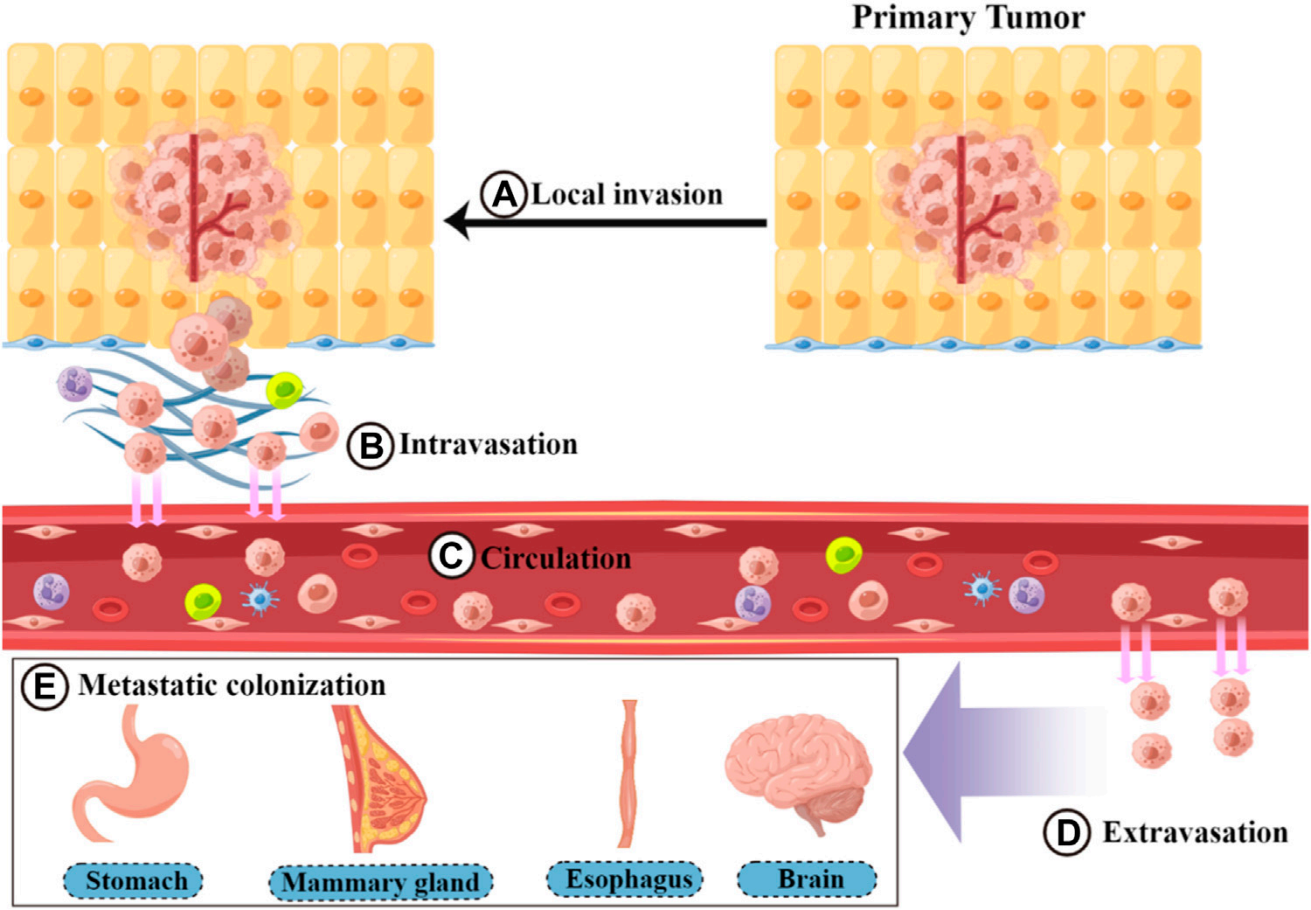
Process of the invasion–metastasis cascade. The invasion–metastasis cascade is complex and includes the following steps: **(A)** local invasion, **(B)** intravasation, **(C)** circulation, **(D)** extravasation, and **(E)** metastatic colonization.

Metastasis is a basic feature of malignant tumors (Liao et al., 2021; Zhou et al., 2021). During this process, tumor cells spread from the primary site of the tumor to the whole body through circulatory channels (lymphatic vessels and blood vessels) or body cavities. Among these, blood circulation is the main route of metastasis (Brabletz, 2012; Welch and Hurst, 2019). Tumor metastasis requires the completion of a complex multistep process collectively referred to as the invasion–metastasis cascade (Valastyan, 2011; Massagué and Ganesh, 2021) (Figure 1). This cascade includes the following five steps: 1) Local invasion: Tumor cells detach from the surrounding cells and primary tumor. This process involves degrading and destroying the extracellular matrix (ECM), mainly the basal membrane, and then invading the surrounding normal tissue (Friedl, 2003; Novikov et al., 2021). 2) Intravasation: Local invasion of tumor cells from the growth site into the lymphatic vessels or vascular cavity, mainly through blood circulation (Zavyalova, 2019). 3) Circulation: Circulating tumor cells (CTCs) entering the bloodstream encounter multiple stresses (such as attacks by NK cells) and adapt and survive. CTCs then circulate through the bloodstream to distant organ sites (Massagué and Obenauf, 2016; Dianat-Moghadam, 2021). 4) Extravasation: CTCs that reach the target organ are captured and grow within the lumen of the blood vessels. Eventually, they break through the vessel wall and enter the target tissue parenchyma (Strilic, 2017; Cheng, 2021). 5) Metastatic colonization: Extravasated disseminated tumor cells adapt to survive in a microenvironment that is different from that of the primary tumor. Most cells are in a dormant state and completely colonize the site (Neophytou et al., 2019; Klein, 2020; Jehanno, 2022). The epithelial–mesenchymal transition (EMT) is an important mechanism by which tumor cells overcome invasion barriers, such as the endothelium (Nieto, 2016). EMT is mainly the result of specific transcription factors (including ZEB and TWIST) that inhibit the expression of epithelial substances (such as E-cadherin) and cause tumor cells to lose intercellular adhesion and detach. In addition, these cytokines increase the expression of mesenchymal components, such as N-calmodulin (Dongre and Weinberg, 2019; Yang et al., 2020; 2021a). EMT plays a facilitating role at the beginning of metastasis by enhancing the invasive ability of tumor cells (Nieto, 2016). In addition to the complete epithelial form (E) and complete mesenchymal form (M), there is an intermediate form of epithelial–mesenchymal plasticity during EMT (Yang et al., 2020; 2021a). However, for extravasated tumor cells to grow and colonize, they need to undergo an epithelial-mesenchymal transition (MET) process that promotes the expression of E-calmodulin (Padmanaban et al., 2019; Bakir et al., 2020; O’Driscoll, 2020). In addition to the autonomous mechanism of tumor cells, the predominant factor influencing tumor metastasis is the tumor microenvironment (TME) (Quail and Joyce, 2013; Taki et al., 2021). The TME consists of tumor cells and non-tumor components, such as the ECM and immune cells surrounding the tumor cells (Arneth, 2019). In most malignant tumors, the TME promotes the growth of tumor cells and enhances their ability to metastasize (Ren et al., 2018; Deepak, 2020). Cancer-associated fibroblasts (CAFs) secrete cytokines and growth factors, such as transforming growth factor-β (TGF-β). These cytokines and growth factors promote mesenchymal transition remodeling of the ECM or directly promote tumor cell proliferation, thereby enhancing tumor cell invasion and metastasis (Kalluri, 2016; Erdogan and Webb, 2017). Tumor-associated macrophages (TAMs) affect almost every step of metastasis, enhancing the metastatic function of the tumor (Lin et al., 2019; Pan, 2020). For example, TAMs can secrete protein hydrolases to degrade the ECM and facilitate tumor cell invasion (Dykes et al., 2019; Jakoš, 2019). They can also drive angiogenesis to promote endocytosis (Fu, 2020). Metastasis is a complex and lengthy disease process, with each step affected by factors in the microenvironment, including the survival pressure during circulation (Psaila and Lyden, 2009; Peinado et al., 2017). Metastasis can be completed only when tumor cells adapt to these unfavorable factors (Coban et al., 2021).

Ferroptosis is a novel form of regulated cell death proposed by Dixon et al., in 2012 (Dixon et al., 2012). The occurrence of ferroptosis is mainly related to the balance between the oxidative and antioxidant systems. The main mechanism is abnormal cell membrane lipid metabolism catalyzed by an excess of ferrous ions. Excessive accumulation of membrane reactive oxygen species (ROS) leads to membrane lipid peroxidation, which causes membrane damage and induces cell death (Yang and Stockwell, 2016; Jiang X. et al., 2021). Iron overload can induce ferroptosis by generating ROS through the Fenton reaction and increasing the activity of oxidation-related enzymes. Iron metabolism is closely related to ferroptosis (Chen, 2020). For example, transferrin receptors can increase iron uptake to promote ferroptosis (Wu et al., 2019; Lu et al., 2021), whereas solute carrier family 40 membrane 1 (SLC40A1) can promote iron release and inhibit ferroptosis (Li, 2021). Thus, factors that reduce iron accumulation, such as iron chelators, can inhibit ferroptosis (Chen et al., 2020). Lipid peroxidation induced by the inhibition of antioxidant systems (mainly GPX4 and system Xc-) is an important contributor to ferroptosis (Kuang, 2020; Tang et al., 2021). GPX4 uses reduced glutathione (GSH) as a substrate to catalyze the reduction of lipid peroxides; it can convert OOH-PE to OH-PE, which directly reduces the accumulation of lipid peroxides, thus inhibiting the occurrence of ferroptosis (Ursini, 2020; Chen et al., 2021c). Therefore, the downregulation of GPX4 expression or decreased activity is an important mechanism of ferroptosis. Small-molecule compounds, such as GPX4 inhibitors, ras selective lethal 3 (RSL3), and ML162, can inhibit GPX4 activity, leading to the accumulation of fatty acid radicals and ultimately triggering ferroptosis (Conrad and Pratt, 2020). The rate-limiting step in the synthesis of GSH as a cofactor of GPX4 is the uptake of cysteine 2 (Cys2) (Ursini, 2020). The Glu/Cys2 reverse transporter of the Xc-system consists of transmembrane transporter solute carrier family 7 member 11 (SLC7A11) and transmembrane regulatory protein solute carrier family 3 member 2 (SLC3A2), which can transfer extracellular Cys2 to intracellularly generate GSH (Liu, 2021a; Liu, 2021c). Therefore, an imbalance in the Xc-system is an important mechanism of ferroptosis. Small molecules such as erastin can inhibit Glu/Cys2 reverse transporter and thus reduce GSH synthesis, leading to a decrease in GPX4 activity and triggering ferroptosis (Wang et al., 2020b). As research progresses, the role of ferroptosis in a variety of diseases is being uncovered. In particular, the regulation of pathological processes in cancer has attracted extensive attention (Chen et al., 2021d).

CircRNAs are circular single-stranded RNAs formed by covalent closure; they are stable, conserved, and widely present across species (Memczak et al., 2013; Patop et al., 2019; Zhou et al., 2020). In addition, circRNAs are characterized by their abundance, diversity, and tissue specificity (Kristensen et al., 2019; Liu, 2022a). CircRNAs can generally be classified into three categories: exon–loop RNA (ecRNA), exon–intron loop RNA (EIcircRNA), and loop–intron RNA (ciRNA) (Zhang, 2013; Li et al., 2015; Liu, 2022a). CircRNAs can regulate gene expression and play important roles in transcription, shearing, and other aspects in a variety of ways, such as by interacting with miRNAs and binding to proteins (Kristensen et al., 2019; Chen and Shan, 2020; Zhou et al., 2020). In addition, circRNAs can affect tumor progression, and studies have reported their abnormal expression in a variety of cancers (Lei et al., 2020; Chen C. et al, 2021; Kristensen, 2022), such as gastric cancer (GC) (Chen D.-L. et al., 2021; Zhang Y. et al, 2021) and colorectal cancer (Shang et al., 2020; Chen D.-L. et al, 2021). Therefore, circRNAs hold potential as disease markers and therapeutic targets. In this review, we focus on ferroptosis-related circRNAs and discuss their functions in tumor metastasis.

## 2 Ferroptosis

### 2.1 Role of ferroptosis in tumor metastasis

**FIGURE 2:**
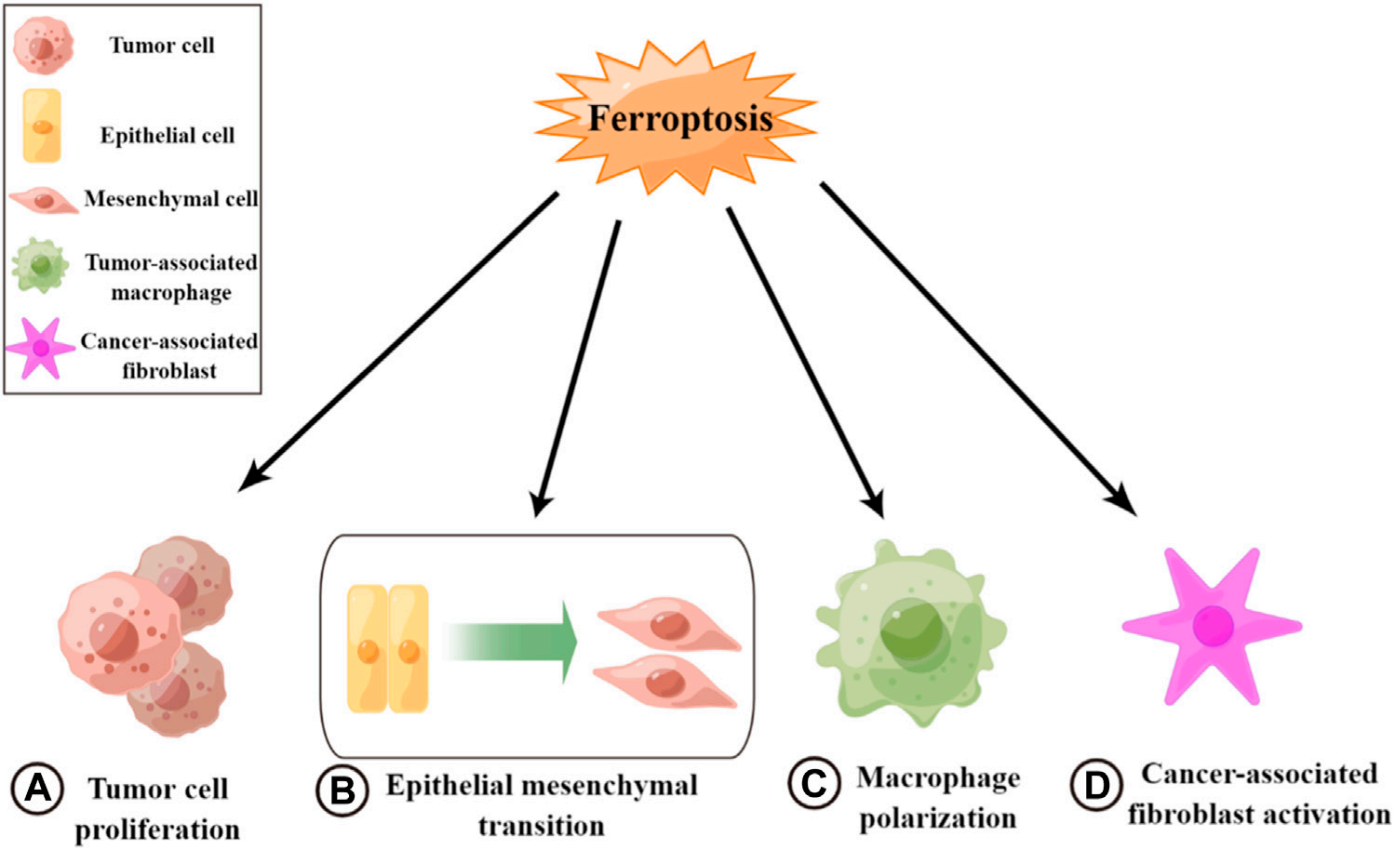
Roles of ferroptosis in tumor metastasis. **(A)** Inhibiting tumor cell growth and cell proliferation. **(B)** Inhibiting epithelial–mesenchymal transition. **(C)** Promoting macrophage polarization toward M1. **(D)** Inactivating cancer-associated fibroblasts.

Ferroptosis plays an inhibitory role in tumor metastasis and suppresses tumor progression, mainly affecting both tumor cells and the TME (Figure 2). In tumor cells, a variety of drugs can induce the onset of ferroptosis and inhibit tumor cell growth and proliferation. Thus, they can weaken the metastatic ability of tumors and exert antitumor effects (Su, 2020). For example, curcumin inhibits tumor growth by activating autophagy-induced ferroptosis, whereas metformin inhibits breast cancer growth by inhibiting SLC7A11-induced ferroptosis (Yang et al., 2021c). In addition, some enzymes and drugs can induce the onset of ferroptosis and inhibit EMT to reduce tumorigenesis, such as glyceric acid in melanoma (Wang, 2020) and functional deacetylase 3 in gallbladder cancer (Liu, 2021b). However, studies have also suggested the opposite, indicating that there are some common changes in ferroptosis and EMT and that these processes are positively correlated in certain diseases (Sun, 2021). Additionally, ferroptosis may have a facilitative effect on EMT (Yao, 2022). Thus, the mechanism between these two processes needs to be further explored (Ebrahimi, 2022). Ferroptosis can also inhibit TME remodeling and affect the activity of tumor-associated cells to exert antitumor effects. Ferroptosis and ferroptosis-related genes can promote macrophage polarization toward M1 by affecting TAM polarization. Macrophages enhance their pro-inflammatory and antitumor capacities and inhibit tumor cell migration (Gu, 2021; Zhou et al., 2022). Some antitumor agents and cytokines can induce ferroptosis to inactivate CAFs and inhibit tumor metastasis, such as disulfiram/copper in nasopharyngeal carcinoma (Li Y. et al., 2020). Therefore, the induction of ferroptosis may be an effective measure for inhibiting tumor metastasis.

### 2.2 Regulation of ferroptosis by circRNAs

**FIGURE 3:**
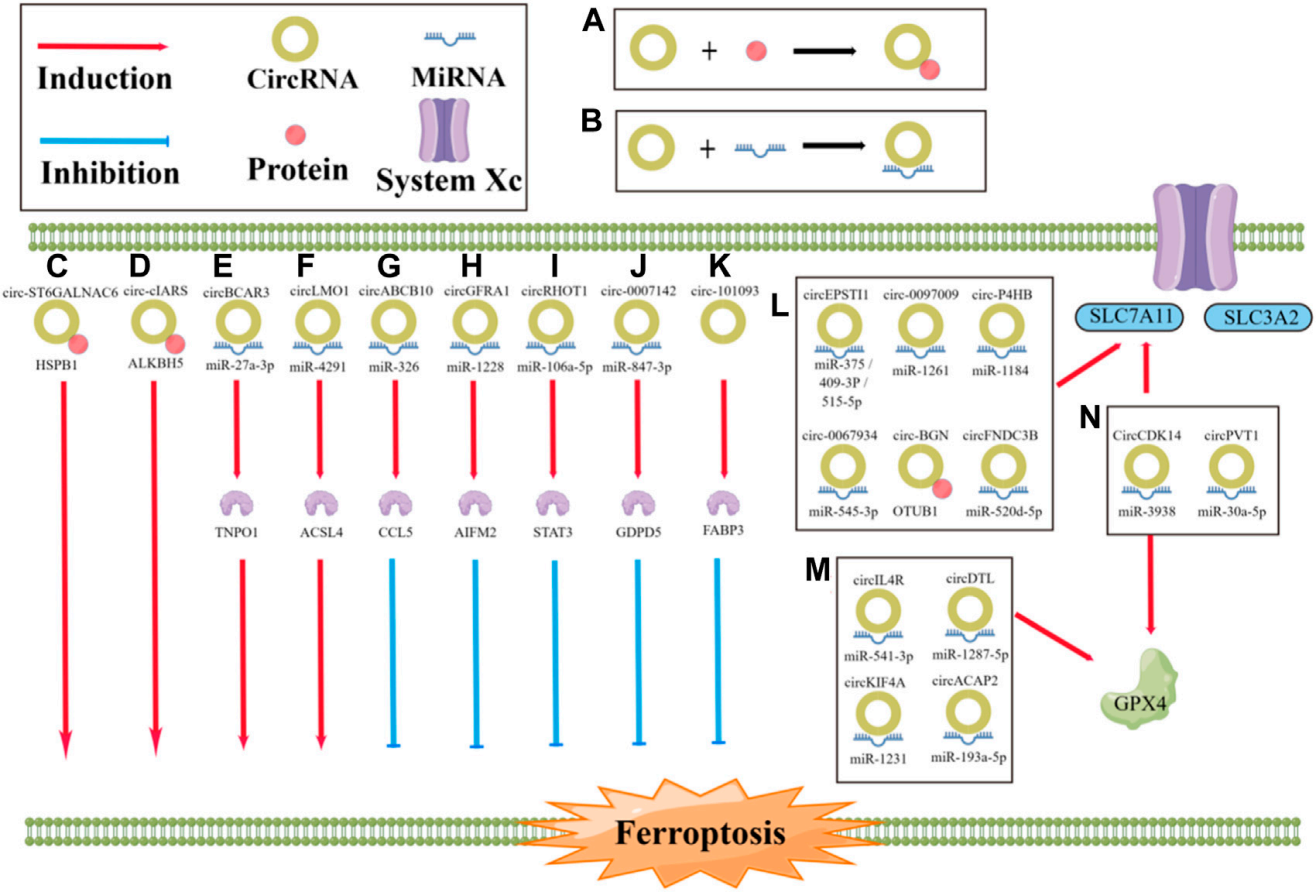
Role of circRNA in the regulation of ferroptosis. **(A, B)** The progression of ferroptosis can be influenced by circRNAs, either by sponging miRNA or by directly binding to proteins, which can promote or inhibit the process; **(C)** circST6GALNAC6 directly binding to HSPB1 to promote the ferroptosis; **(D)** circ-cIARS directly binding to ALKBH5 to promote the ferroptosis; **(E)** circBCAR3 sponging miR-27a-3p to promote the ferroptosis; **(F)** CircLMO1 sponging miR-4291 to promote the ferroptosis; **(G)** circABCB10 sponging miR-326 to inhibit the ferroptosis; **(H)** circGFRA1 sponging miR-1228 to inhibit the ferroptosis; **(I)** circRHOT1 sponging miR-106a-5p to inhibit the ferroptosis; **(J)** circ0007142 sponging miR-847-3p to inhibit the ferroptosis; **(K)** circRNA-101093 directly binding to FABP3 to inhibit the ferroptosis; **(L, M)** CircRNAs sponging miRNA or directly binding to proteins to regulate the ferroptosis.

CircRNAs and ferroptosis are both closely related to cancer development and metastasis; however, the links and mechanisms between them are not well understood (Zhi, 2021; Zuo et al., 2022). By reviewing relevant studies, we summarize the effects of specific circRNAs in cancer on the process of ferroptosis, which have been found to either promote or inhibit ferroptosis (Figure 3; Table 1). Regarding inhibition, in terms of the antioxidant system, circ0097009 (Lyu et al., 2021), circP4H (Pan et al., 2022), circBGN (Wang et al., 2022), circFNDC3B (Yang et al., 2021b), and other circRNAs can sponge or repress miRNAs to promote SLC7A11 expression and thus inhibit the occurrence of ferroptosis (Wang H. H. et al., 2021; Wu et al., 2021; Yao, 2021; Chen et al., 2022). Similar to SLC7A11, circRNAs can also inhibit ferroptosis through miRNA-targeted upregulation of GPX4 (Xu et al., 2020; Chen, 2021c; Shanshan, 2021; Liu, 2022b), such as the “circIL4R/miR-541-3p/GPX4” axis (Xu et al., 2020) and “circDTL/miR-1287-5p/GPX4” axis (Shanshan, 2021). CircRNAs can also affect the metabolism of unsaturated fatty acids by reducing lipid peroxidation. For example, circRNA-101093 can promote the transport of arachidonic acid with fatty acid binding protein 3 (FABP3) to produce N-arachidonyl taurine, thus inhibiting ferroptosis (Zhang, 2022). In addition, circRNAs can bind to miRNAs to regulate ferroptosis-related genes at both the mRNA and protein levels. For example, circGFRA1 can attenuate the activity of miR-1228, promote the expression of the ferroptosis inhibitory factor apoptosis-inducing factor mitochondrial 2 (AIFM2), and inhibit ferroptosis (Bazhabayi et al., 2021). In addition, the miR-847-3p/GDPD5 axis of circ0007142 (Wang Y. et al, 2021) and miR-326/CCL5 axis of circABCB10 (Xian, 2020) also have this mechanism (Zhang H. M. et al, 2021; Zhang Y. et al, 2021). In contrast to repression, circRNAs can also directly regulate the expression of ferroptosis-related genes through miRNAs. CircLMO1 may promote the expression of acyl-CoA synthetase long-chain family member 4 (ACSL4) through miR-4291 to increase its mRNA and protein levels and promote ferroptosis (Ou et al., 2022). CircRNAs can also indirectly regulate ferroptosis-related genes via regulating the expression of P38. For example, circST6GALNAC6 can activate the P38/MAPK signaling pathway through the HSPB1/P38 axis, which, in turn, regulates ferroptosis-associated genes and indirectly promotes ferroptosis (Wang et al., 2020a). In addition, circRNAs can promote ferroptosis by affecting other molecules through miRNAs. For example, circBCAR3 binds to miR-27a-3p to increase the expression level of transportin-1, which, in turn, promotes ferroptosis (Xi et al., 2022). Circ0000190 promotes ferroptosis by regulating zinc and ring finger 3 through miR-382-5p (Jiang et al., 2022). CircRNAs can also bind directly to proteins to regulate ferroptosis. For example, cIARS can bind to the RNA binding protein (RBP) ALKBH5 and inhibit its function to promote ferroptosis (Liu, 2020). As research continues, increasing attention is being paid to the role of circRNAs and ferroptosis in cancer. Understanding and exploring the links between circRNAs, ferroptosis, and cancer and the mechanisms involved will help provide new therapeutic measures.

**TABLE 1:** Role of ferroptosis-related circRNA in cancers.

circRNA	Cancers	Regulatory way	Function	Ref
circ-0097009	Hepatocellular carcinoma	miR-1261/SLC7A11	Inhibiting ferroptosis	[86]
circIL4R		miR-541-3p/GPX4	Inhibiting ferroptosis	[120]
circ-cIARS		ALKBH5(RBP)	Inducing ferroptosis	[107]
circ-P4HB	Lung adenocarcinoma	miR-1184/SLC7A11	Inhibiting ferroptosis	[87]
circ-101093		FABP3/AA/NAT/ACSL4, LPCAT3 and PLTP	Inhibiting ferroptosis	[160]
circEPSTI1	Cervical cancer	miR-375/409-3P/515-5p/SLC7A11	Inhibiting ferroptosis	[91]
circACAP2		miR-193a-5p/GPX4	Inhibiting ferroptosis	[97]
circLMO1		miR-4291/ACSL4	Inducing ferroptosis	[108]
circPVT1	Esophageal cancer	miR-30a-5p/FZD3/p-β-catenin, GPX4, and SLC7A11	Inhibiting ferroptosis	[93]
circBCAR3		miR-27a-3p/TNPO1	Inducing ferroptosis	[105]
circ-BGN	Breast cancer	OTUB1/SLC7A11	Inhibiting ferroptosis	[88]
circGFRA1		miR-1228/AIFM2	Inhibiting ferroptosis	[100]
circRHOT1		miR-106a-5p/STAT3	Inhibiting ferroptosis	[102]
circ-0067934	Thyroid cancer	miR-545-3p/SLC7A11	Inhibiting ferroptosis	[90]
circCDK14	Glioma	miR-3938/PDGFRA/SLC7A11 and GPX4	Inhibiting ferroptosis	[92]
circFNDC3B	Oral squamous cell carcinoma	miR-520d-5p/SLC7A11	Inhibiting ferroptosis	[89]
circDTL	Non-small cell lung cancer	miR-1287-5p/GPX4	Inhibiting ferroptosis	[96]
circKIF4A	Papillary thyroid cancer	miR-1231/GPX4	Inhibiting ferroptosis	[94]
circ-0007142	Colorectal cancer	miR-847-3p/GDPD5	Inhibiting ferroptosis	[101]
circABCB10		miR-326/CCL5	Inhibiting ferroptosis	[102]
circ-ST6GALNAC6	Bladder cancer	HSPB1/P38	Inducing ferroptosis	[104]
circ-0000190	Gastric cancer	miR-382-5p/ZNRF3	Inducing ferroptosis	[106]

## 3 Effect of ferroptosis-related circRNAs on tumor metastasis

CircRNAs can not only regulate miRNAs in the circRNA–miRNA–mRNA network but also interact with proteins and even play a role in the occurrence and development of cancer as exosomes. Exosomal circLPAR1 and methyltransferase-like 3 (METTL3) competitively bind RBP eIF3h, effectively reducing the translation of brominated protein 4 (BRD4) and subsequently inhibiting the proliferation, invasion, and migration of colorectal cancer cells (Zheng et al., 2022). Moreover, the secretion of circUHRF1 in exocrine form can promote the progression of hepatocellular carcinoma (HCC) by degrading miR-3c-449p in NK cells and upregulating the expression of TIM-3, thereby inhibiting NK cell function (Zhang et al., 2020). However, ferroptosis-related circRNAs can sponge with miRNAs to regulate the tumor metastasis. Some miRNAs are abnormally expressed in tumor tissue, promoting or inhibiting malignant behavior in tumors by modulating their downstream signaling pathways (Huang et al., 2020). Competitive endogenous RNA (ceRNA) can be mutually modulated at the post-transcriptional level by competing with shared miRNAs (Qi et al., 2015). CircRNAs contain miRNAs response elements, enabling them to act as ceRNAs and compete with downstream targets to modulate their downstream pathways (Xiong et al., 2018). These circRNAs bind to miRNAs to modulate their downstream targets, which may promote or inhibit tumor metastasis.

## 4 Ferroptosis-related circRNAs can sponge with miRNA to regulate the metastasis in cancers

### 4.1 Digestive system tumors

In the mechanism of tumor development and metastasis in the digestive system, ferroptosis-related circRNAs interacts with miRNA to regulate downstream target substances and changes GSH content through the Xc-system to affect GPX4 or directly change GPX4 activity. This, in turn, creates an imbalance in the antioxidants of tumor cells, thus regulating the occurrence or inhibition of ferroptosis in tumor cells. This mechanism occurs in several digestive system tumors, including HCC, GC, oral squamous cell carcinoma (OSCC), and esophageal squamous cell carcinoma (ESCC).

Lyu et al. (Lyu et al., 2021) found that circ0097009 was upregulated in HCC, and knockdown of circ0097009 inhibited the growth and invasion of HCC cells, indicating that circ0097009 plays an important role in the progression of HCC. Further research revealed that circ0097009 functionally interacts with miR-1261 and acts as a ceRNA by sponging miR-1261, thereby modulating SLC7A11, the downstream target gene of miR-1261. The researchers further found that the knockdown of circ0097009 reduced SLC7A11, a component of the Xc-system identified as a key regulator of ferroptosis in cancer cells. Thus, the knockdown of circ0097009 promotes ferroptosis through the circ0097009/miR-1261/SLC7A11 axis. In addition, the knockdown of circ0097009 led to a decrease in the GSH/GSSG ratio, leading to the inactivation of GPX4, another key regulator of ferroptosis that reduces lipid peroxides. Thus, the knockdown of circ0097009 promotes ferroptosis in HCC cells through two independent pathways, system Xc- and GPX4, thereby inhibiting the growth and invasion of HCC cells. In addition, Xu et al. (Xu et al., 2020) identified upregulated circIL4R in HCC tissues and cell lines, and the knockdown of circIL4Rd inhibited cell growth and promoted ferroptosis in HCC cells. CircIL4R acts as a molecular sponge of miR-541-3p in HCC cells and exerts positive regulation on GPX4 in hepatocytes by sponging miR-541-3p. Moreover, the authors further experimentally demonstrated that downregulating miR-541-3p alleviates the inhibition of tumorigenesis and promotion of ferroptosis caused by circIL4R knockdown, and inhibiting circIL4R reduces GPX4 by upregulating miR-541-3p to prevent tumor cell proliferation and inhibit HCC tumor progression, thereby hindering HCC growth and metastasis *in vivo*.

In the GC, Jiang et al. (Jiang et al., 2022) found that circ0000190 acts as a sponge for miR-385-5P to target ZNRF3 to inhibit GC cell proliferation and motility and promote cell death. They found that circ0000190 was downregulated in GC tissues and cell lines, whereas overexpressed circ0000190 inhibited the proliferation, migration, and invasion of GC cells and promoted erastin- or RSL3-mediated ferroptosis. miR-382-5p is the target of circ0000190, and ZNRF3 is the target of miR-382-5p. Circ0000190 inhibits GC progression by acting as a miR-382-5p sponge; miR-382-5p accelerates the proliferation and transfer of GC cells and inhibits ferroptosis by regulating ZNRF3. In addition, they found that circ0000190 overexpression inhibited the growth of xenograft tumors *in vivo*.

Among oral and esophageal tumors, OSCC is a widespread head and neck malignancy of the oropharynx and oral cavity (Panarese et al., 2019). OSCC is life-threatening cancer, occurring in 8.0%–8.5% of men and 4.0%–8.1% of women (Solomon et al., 2018). Cyclic RNAs play various roles in OSCC pathogenesis (Zhang et al., 2019). Yang et al. found that circFNDC3B as a ceRNA of miR-520d-5p induced SLC7A11 expression through the circFNDC3B/miR-520d-5p/SLC7A11 axis, which is involved in the regulation of ferroptosis-related phenotypes in OSCC cells and development of OSCC (Yang et al., 2021b). Previous studies have demonstrated the critical function of circFNDC3B in cancer development; for example, it promotes invasion and migration by regulating the expression of CD44 and E-cadherin in GC cells (Hong et al., 2019). Previous studies have also reported the function of miR-520d-5p in cancer development, where it inhibits metastasis and tumor growth by targeting CTHRC1 in colorectal cancer (Yan et al., 2015). SLC7A11 is a key regulator of ferroptosis and metabolism (Lin et al., 2020; Mukhopadhyay et al., 2021), and miR-375/SLC7A11 signaling regulates OSCC cell proliferation and invasion (Wu et al., 2017). Therefore, circFNDC3B protects OSCC cells from tincture, promotes OSCC progression by modulating the miR-520d-5p/SLC7A11 axis, and may also affect the invasion and movement of OSCC cells. Moreover, circFNDC3B regulates cancer cell proliferation, apoptosis, and migration in a variety of malignancies, such as esophageal, bladder, and colon cancers (Liu et al., 2018; Luo et al., 2018; Pan et al., 2020).

Esophageal cancer (EC), originating from esophageal epithelial cells (Sung et al., 2021), includes two main pathological types: esophageal adenocarcinoma and ESCC (Smyth et al., 2017). CircRNAs play a crucial role in the pathogenesis and progression of ESCC by participating in a variety of regulatory mechanisms, such as ceRNAs, protein interactions, and the regulation of gene transcription and translation in ESCC cells (Feng et al., 2020). Circ0087378 inhibits the tumorigenesis and progression of ESCC by acting as a competitive endogenous RNA and upregulating E2F3 expression (Wang J. et al., 2020). Moreover, circPVT1 regulates the chemosensitivity of ESCC cells through ferroptosis and Wnt/β-catenin pathways via miR-30a-5p/FZD3. The Frizzled (FZD) family is a transmembrane receptor of the Wnt signaling pathway with 10 subtypes (FZD1–10) (Bhanot et al., 1996). FZD3 promotes the proliferation and invasion of cancer cells through the Wnt/β-catenin pathway (Qiao et al., 2017; Xia et al., 2018; Li et al., 2019). In addition, Zhong et al. (Zhong et al., 2019) demonstrated thatcircPVT1 enhances the malignant phenotype of ESCC by modulating the miR-4663/Pax and PPAR axes, including proliferation and invasion. Xi et al. (Xi et al., 2022) reported that CircBCAR3 is significantly upregulated in EC tissues and cells, and the knockdown of circBCAR3 inhibits the proliferation, migration, invasion and ferroptosis of EC cells; Further functional studies found that circBCAR3 can act as a ceRNA to increase the expression of Transportin-1 (TNPO1) via sponging with miR-27a-2p, thereby contributing to the progression of EC.

### 4.2 Reproductive system tumors

Among reproductive system tumors, cervical cancer is the fourth most common malignancy in the world and the fourth leading cause of cancer death in women, with extremely high morbidity and mortality rates (Sung et al., 2021). CircRNAs play a key role in tumorigenesis and tumor progression; however, the biological effects of most circRNAs on cervical cancer remain unclear. As a novel form of cancer-related cell death, ferroptosis is closely associated with cervical cancer progression (Jiang X. F. et al., 2021; Qi et al., 2021; Wu et al., 2021). Ou et al. (Ou et al., 2022) demonstrated that circLMO1 inhibited the growth and metastasis of cervical cancer by triggering miR-4291/ACSL4-mediated cellular ferroptosis. CircLMO1 levels were downregulated in cervical cancer tissue, and circLMO1 overexpression significantly inhibited the growth and metastasis of cervical cancer cells *in vivo*. CircLMO1 silencing promoted the proliferation and invasion of cervical cancer cells. To explore the related cellular molecular mechanisms, the authors further explored the death pattern induced by circLMO1, and the results showed that it promoted cervical cancer cell death by triggering ferroptosis and apoptosis. In addition, circLMO1 was an important mode of cervical cancer cell death, which was observed by monitoring the response of circLMO1 to the effects of erastin (an activator of ferroptosis) in cervical cancer cells. Thus, by regulating ferroptosis, circLMO1 accelerates cervical cancer cell death.

### 4.3 Endocrine system tumors

In endocrine system tumors, including thyroid and breast cancer, ferroptosis-related circRNAs can regulate tumor metastasis by acting as sponges for miRNAs.

Based on cancer statistics worldwide, thyroid cancer is the fifth most common malignancy (Siegel et al., 2016). There are various subtypes of thyroid cancer, and 85%–90% are papillary thyroid cancers (Santiago et al., 2020). Approximately 90% of patients can be treated with standard treatments (Park et al., 2020). However, locoregional recurrences or distant metastases occur in almost 10% of thyroid carcinoma cases, which remains a challenge in the treatment of thyroid cancer (Pelizzo et al., 2008). Chen et al. found that the knockdown of circKIF4A could increase GPX4 expression through the circKIF4A/miR-1231/GPX4 axis, leading to thyroid metastasis via inhibition of cancer cell ferroptosis (Chen W. K. et al., 2021). Thus, circRKIF4A could inhibit ferroptosis to promote thyroid tumor progression. Circ0067934 upregulates SLC7A1 expression via the miR-545-3p/SLC7A11 axis, which inhibits thyroid cancer cell ferroptosis and promotes thyroid cancer growth and metastasis (Wang et al., 2017; Wang H. H. et al., 2021; Ji et al., 2022). Thus, targeting this regulatory axis or blocking the function of circ0067934 could potentially be a therapeutic strategy to combat thyroid cancer.

Breast cancer is the most common fatal cancer in women worldwide, and despite advances in various treatments, it remains the second leading cause of tumor-related death in women (Rodgers et al., 2017). CircRNAs play an important role in modulating breast cancer progression (Li S. Q. et al., 2020; Wang R. J. et al., 2020). For example, circABCB10 promotes the progression and proliferation of breast cancer cells by targeting miR-1271 (Liang et al., 2017). In addition, circRHOT1 is abnormally expressed in cancer cells, promoting proliferation, migration, and invasion and inhibiting apoptosis (Qu et al., 2019). CircRHOT1 promotes breast cancer cell proliferation and inhibits the apoptosis of breast cancer cells, enhancing their invasion and migration (Zhang H. M. et al., 2021). To evaluate the role of circRHOT1 in myelitis, Zhang et al. analyzed the effects of circRHOT1 on elastin-induced inhibition of cell growth, intracellular ROS, iron levels, and the expression of GPX4 and SLC7A11, which are markers of cellular ferroptosis. They found that circRHOT1 can reduce ferroptosis in breast cancer cells. It was later demonstrated that circRHOT1 induces ferroptosis by targeting signal transducer and activator of transcription 3 (STAT3) by sponging miR-106a-5p, thereby enhancing the invasion and metastasis of breast cancer cells.

To improve targeted therapy, breast cancer is clinically divided into three main subtypes according to immunohistochemical markers: estrogen receptor-positive breast cancer, triple-negative breast cancer, and human epidermal growth factor receptor 2 (HER-2)-positive breast cancer. HER-2-positive tumors account for 20% of all breast cancers (Yang et al., 2015). Tumors with elevated HER-2 levels have a high rate of tumor growth and are aggressive; therefore, the *HER-2* gene plays an important role in tumor biology (Agazie and Hartman, 2019). Bazhabayi et al. (Bazhabayi et al., 2021) report that circGFRA1 was upregulated in HER-2-positive breast cancer cells and tissues, and silencing circGFRA1 inhibited the proliferation of HER-2-positive breast cancer cells while inhibiting their infiltration and metastasis. Furthermore, CircGFRA1 is predominantly present in the cytoplasm, suggesting that it can intersect with most miRNAs located in the cytoplasm, promoting the malignant progression of HER-2-positive breast cancer by acting as a sponge for miR-1228 and enhancing AIFM2 expression.

### 4.4 Nervous system tumors

Glioma is a highly prevalent and aggressive malignancy of the central nervous system, with a poor prognosis. Despite the availability of advanced chemotherapy, radiation therapy, and surgery, patients with glioma have poor median survival rates. In addition to accelerated proliferation, invasiveness, and treatment resistance, the poor prognosis of severe glioma stems from a limited understanding of the potential pathways as well as a lack of early diagnosis and effective treatments (Lapointe et al., 2018). The circRNA/miRNA/mRNA pathway is critical for the development and progression of gliomas. Overexpression of circHIPK3 enhances the proliferation and invasion ability of glioma cells by chelating miR-124-3p and increasing STAT3 levels (Hu and Zhang, 2019). Chen et al. (Chen et al., 2022) identified circRNAs in glioma by RNA-Seq analysis, detecting 158 circRNAs with high expression, 1,090 relatively low-expression circRNAs relative to non-tumor brain tissue, and seven downregulated and five upregulated circRNAs using qRT-PCR. Because the parent gene *CDK14* of circCDK14 is a cell cycle-dependent kinase involved in the occurrence and development of gliomas, further examination of the level of circCDK14 in gliomas revealed that the level of circCDK14 in human glioma cells was significantly increased, and circCDK14 was expressed at higher levels in grade II–IV gliomas than grade I–II gliomas. CircCDK14 is predominantly expressed in the cytoplasm. The author further evaluated the biological activity of circCDK14 in gliomas and found that circCDK14 overexpression significantly increased the proliferation, migration, and invasion of glioma cells *in vitro*. In addition, Kaplan-Meier analysis revealed that circCDK14 levels were negatively correlated with overall survival in patients with glioma. Because circCDK14 is mostly localized in the cytoplasm, the authors speculated that circCDK14 may be a glioma-chelating miRNA. Subsequently, they predicted four miRNAs and showed that miR-3938 and circCDK14 interact in the cytoplasm of glioma cells by luciferase reporter gene testing. CircCDK14 plays a sponging role for miR-3938, and miR-3938 can reverse the biological role of circCDK14 in gliomas. Moreover, PDGFAR was shown to be a direct target gene for miR-3938, and circCDK14 regulated PDGFAR expression by sponging miR-3938. To further verify the role of PDGFRA and circCDK14 in cell ferroptosis, the authos further observed the mitochondrial morphology of glioma cells using transmission electron microscopy, and the results demonstrated that PDGFRA expression was negatively correlated with glioma cell ferroptosis. CircCDK14 can reduce the sensitivity of glioma cells to ferroptosis by regulating PDGFRA expression, thereby promoting the formation and metastasis of gliomas *in vivo*.

## 5 Conclusions and perspectives

Many studies have shown that circRNAs can regulate iron death in tumor tissues and cells and that most circRNAs promote or inhibit the occurrence of iron death and cancer progression by affecting miRNAs, providing potential therapeutic methods for cancer treatment. Certain cancers may be affected by a complex circRNA functional network rather than a single circRNA; therefore, the logical progression is to screen for circRNAs and then study the function of a set of significantly differentially expressed circRNAs or a single circRNA. To reveal the role of circRNAs in tumor progression, their potential targets should be explored in further studies. CircRNAs can not only regulate miRNAs in the circRNA–miRNA–mRNA network but also interact with proteins and even play a role in the development of cancer as exosomes.

Exploring the mechanism of ferroptosis-related circRNAs in tumor metastasis provides alternative pathways and targets for the further exploration of cancer therapies. Ferroptosis is a metabolic form of oxidative stress-induced cell death, and tumor cells are more metabolically active and have higher ROS loads than normal cells; therefore, cancer cells may have a higher tendency toward ferroptosis than normal cells, which has been confirmed by some studies. Initially, the chemical compounds that induce ferroptosis were identified as novel treatments for cancer. Subsequent mechanistic studies have shown that many cancer-related genes and signaling pathways regulate ferroptosis. Some experiments have shown that mesenchymal and dedifferentiated cancer cells, which are generally resistant to apoptosis and common therapies, as well as so-called “therapeutic persistence” cancer cells, are highly sensitive to ferroptosis inducers, further emphasizing the potential of ferroptosis induction as a novel cancer therapy. CircRNAs have diverse functions in the regulation of cancer metastasis. In addition to influencing ferroptosis-related mechanisms, they also play a role in modulating a range of processes, including apoptosis and cancer immunity. This review summarizes the effects of ferroptosis-related circRNAs on cancer metastasis. Although the regulatory mechanisms of these circRNAs are still not fully understood, they provide promising new targets for cancer therapy. Future research should aim to identify the specific mechanisms by which ferroptosis-related circRNAs regulate cancer metastasis in different types of cancers. Notably, circRNAs are only one type of non-coding RNA associated with tumors, and long non-coding RNAs have a much wider range of tumor-related studies. As such, investigating the effects of other non-coding RNAs alongside ferroptosis-related circRNAs may offer new insights into cancer progression. Although studies on ferroptosis-related circRNAs in certain cancers remain limited, there is a strong rationale for further exploration in a wider range of cancer types. Such investigations can drive innovation and potentially uncover new therapeutic options for the treatment of cancer.

## Figures and Tables

**Table:** 

**ACSL4**	Acyl-CoA synthetase long-chain family member 4
**AIFM2**	Apoptosis-inducing factor mitochondrial 2
**ALKBH5**	AlkB Homolog 5
**CAF**	Cancer-associated fibroblasts
**CCL5**	C-C motif chemokine ligand 5
**ceRNA**	Competitive endogenous RNA
**circFNDC3B**	circular RNA FNDC3B
**CircRNA**	Circular RNA
**CTCs**	Circulating tumor cells
**Cys2**	cysteine
**EAC**	esophageal adenocarcinoma
**EC**	Esophageal cancer
**ECM**	Extracellular matrix
**ecRNA**	exon-loop RNA
**EICircRNA**	exon-intron loop RNA
**EMT**	Epithelial mesenchymal transition
**MET**	Epithelial-mesenchymal transition
**ER**	estrogen receptor
**ESCC**	esophageal squamous cell carcinoma
**FABP3**	Fatty acid-binding protein 3
**GC**	gastric cancer
**GDPD5**	Glycerophosphodiester phosphodiesterase domain containing 5
**GPX4**	glutathione peroxidase 4
**HCC**	hepatocellular carcinoma
**HSPB1**	Heat shock protein B1
**OSCC**	oral squamous cell carcinoma
**RBP**	RNA binding protein
**ROS**	reactive oxygen species
**RSL3**	ras selective lethality 3
**SLC3A2**	transmembrane regulatory protein solute carrier family 3 member 2
**SLC40A1**	solute carrier family 40 membrane 1
**SLC7A11**	transmembrane transporter solute carrier family 7 member 11
**STAT3**	Signal transducer and activator of transcription 3
**TAM**	Tumor-associated macrophages
**TFRC**	the transferrin receptor
**TGF-β**	transforming growth factor-β
**TME**	tumor microenvironment
**TNPO1**	Transportin-1
**FABP3**	fatty acid binding protein 3.
